# Radiation-Induced Synthesis of a Minocycline-Derived Polycyclic Scaffold with Anti-Inflammatory and Antibacterial Effects

**DOI:** 10.3390/molecules31030435

**Published:** 2026-01-27

**Authors:** Gyeong Han Jeong, Hanui Lee, Tae Hoon Kim, Byung Yeoup Chung, Seung Sik Lee, Hyoung-Woo Bai

**Affiliations:** 1 Advanced Radiation Technology Institute (ARTI), Korea Atomic Energy Research Institute (KAERI), Jeongeup 56212, Republic of Korea; jkh4598@kaeri.re.kr (G.H.J.); hnlee11@kaeri.re.kr (H.L.); bychung@kaeri.re.kr (B.Y.C.); 2 Department of Food Science and Biotechnology, Daegu University, Gyeongsan 38453, Republic of Korea; skyey7@daegu.ac.kr; 3 Department of Radiation Science, University of Science and Technology (UST), Daejeon 34113, Republic of Korea

**Keywords:** radiation, minocycline, minocyclinosin A, anti-inflammatory, anti-bacterial

## Abstract

Radiation is widely used as a powerful tool for inducing molecular transformation and expanding chemical diversity; however, its application in clinically relevant antibiotics remains limited. Minocycline (**1**), a clinically used tetracycline antibiotic, was subjected to gamma irradiation at doses of up to 30 kGy, resulting in the formation of a previously unreported radiation-induced derivative, minocyclinosin A (**2**). The structure of the newly generated compound was elucidated by comprehensive spectroscopic analyses, including one- and two-dimensional nuclear magnetic resonance spectroscopy and high-resolution electrospray ionization mass spectrometry, which revealed extensive A-ring cleavage, degradation, and recyclization to form a unique cyclopenta[b]anthracene-type tetracycline scaffold. Biological evaluation revealed that minocyclinosin A exhibited enhanced anti-inflammatory activity by suppressing lipopolysaccharide-induced nitric oxide production in RAW 264.7 macrophages, while maintaining antibacterial activity against skin inflammation-associated *Staphylococcus* species. High-performance liquid chromatography further demonstrated a clear dose-dependent molecular conversion, with irradiation at 30 kGy affording minocyclinosin A as the major product with a conversion efficiency of approximately 78.3%.

## 1. Introduction

Tetracyclines are a well-established class of broad-spectrum antibiotics that have been used to treat bacterial infections for several decades [1]. Structurally, tetracyclines are characterized by a polycyclic naphthacene core bearing multiple functional groups, which contribute to their strong antibacterial activity by inhibiting bacterial protein synthesis and binding to the 30S ribosomal subunit [2]. In addition to their antimicrobial effects, tetracyclines have attracted considerable attention for their non-antibiotic biological activities, including anti-inflammatory, immunomodulatory, and matrix metalloproteinase inhibitory effects, thus expanding their therapeutic relevance beyond infectious diseases [3,4].

Minocycline is a semi-synthetic second-generation tetracycline antibiotic that exhibits improved lipophilicity and pharmacokinetic properties compared with earlier tetracyclines [5]. Because of its enhanced tissue penetration, minocycline has been extensively used not only as an antibiotic but also as an anti-inflammatory agent for treating various inflammatory and immune-related disorders [6,7]. Previous studies have demonstrated that minocycline can suppress inflammatory mediators, including nitric oxide (NO) and pro-inflammatory cytokines, highlighting its potential as a multifunctional therapeutic agent [8,9]. In addition to their direct antibacterial effects, tetracycline antibiotics have been widely used in the management of inflammatory skin disorders in which bacterial colonization plays a critical role. *Staphylococcus intermedius* (*S. intermedius*), *S. aureus*, and *S. epidermidis* are representative Gram-positive bacteria closely associated with inflammatory and allergic skin conditions [10,11,12]. *S. aureus* is a major pathogenic bacterium implicated in dermatitis and eczema, while *S. epidermidis*, although a common skin commensal, contributes to chronic inflammation under dysbiotic conditions [13,14]. *S. intermedius* has also been reported to exacerbate inflammatory responses during skin and soft tissue infections [15]. Consequently, compounds that simultaneously modulate inflammatory responses and control skin-associated bacteria are of considerable interest for treating inflammatory skin diseases. However, despite the broad therapeutic potential of tetracycline antibiotics, the generation of structurally novel derivatives using conventional synthetic approaches remains challenging because of their complex polycyclic frameworks and multiple sensitive functional groups.

In this context, gamma irradiation has been used as an alternative and efficient strategy for molecular diversification, enabling chemical transformations that are difficult to achieve using traditional organic syntheses [16]. Gamma irradiation induces highly reactive radical species through solvent ionization, which can trigger a variety of chemical reactions, including bond cleavage, functional group modification, hydrolysis, and cyclization, without the need for additional reagents or catalysts [17]. This approach has been successfully applied to various natural products to generate structurally diverse compounds with enhanced biological activities [17,18,19]. However, most previous studies have primarily focused on polyphenol-derived scaffolds, and the application of radiation-induced molecular transformations to clinically relevant antibiotic compounds remains largely unexplored. Accordingly, this study investigates the feasibility of using gamma irradiation as a one-step molecular transformation strategy for minocycline, a multifunctional tetracycline antibiotic, to identify structurally novel derivatives that retain their antibacterial properties while exhibiting enhanced anti-inflammatory activity.

## 2. Results and Discussion

### 2.1. Isolation and Characterization of the Newly Generated Product, Minocyclinosin A

**Figure 1:**
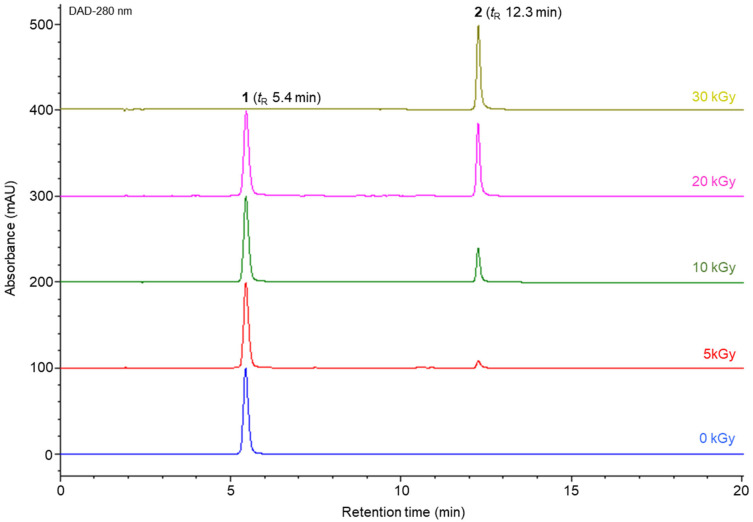
HPLC chromatograms of minocycline after γ-irradiation at different doses: 0 (blue), 5 (red), 10 (green), 20 (pink), and 30 kGy (yellow). Analytical conditions are described in the Materials and Methods section. Peak **1**, minocycline; peak **2**, minocyclinosin A.

The gamma irradiation-induced transformation of minocycline was monitored using high-performance liquid chromatography (HPLC) at 280 nm (Figure 1). In the nonirradiated sample (0 kGy), minocycline (peak **1**) was detected as a single major peak with a retention time (*t*_R_) of 5.4 min. Upon gamma irradiation at 5 kGy, a new minor peak (peak **2**) appeared at 12.3 min, indicating the initial formation of the newly generated product. Meanwhile, the parent minocycline peak remained predominant. As the irradiation dose was increased to 10 and 20 kGy, the intensity of minocycline peak **1** progressively decreased, accompanied by a corresponding increase in the newly generated peak **2**, demonstrating a clear dose-dependent conversion. Notably, the minocycline peak was barely detectable in the sample irradiated at 30 kGy, whereas peak **2** became the dominant component in the chromatogram, indicating the near-complete transformation of minocycline into the newly formed compound under high-dose gamma irradiation. Accordingly, to elucidate the structure of the gamma irradiation-induced transformation product, a minocycline sample irradiated at 30 kGy was subjected to chromatographic separation. The crude irradiated mixtures were fractionated by column chromatography using Toyopearl HW-40 and YMC ODS gels as the stationary phases. Based on HPLC-guided purification, the newly generated compound (**2**) was successfully isolated with a purity greater than 95% (Figure S1).

**Figure 2:**
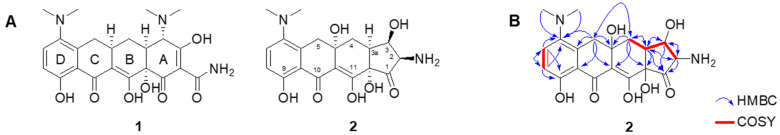
Structures and key 2D NMR correlations of the newly generated product **2** obtained from γ-irradiated minocycline. (**A**): Chemical structures of minocycline (**1**) and minocyclinosin A (**2**). (**B**): Key HMBC and ^1^H-^1^H COSY correlations of compound **2**.

Compound **2** was obtained as a yellowish amorphous powder with the molecular formula C_19_H_22_O_7_N_2_, as determined by high-resolution electrospray ionization mass spectrometry (HRESIMS) (*m*/*z* 413.1336 [M + Na]^+^; calculated for C_19_H_22_O_7_N_2_Na, 413.1319). Its ultraviolet (UV) spectrum showed a maximum absorption peak at 265 nm, indicating a tetracycline backbone [20]. The ^1^H nuclear magnetic resonance (NMR) spectrum of **2** exhibited an *ortho*-coupled aromatic proton system at *δ*_H_ 7.50 (1H, d, *J* = 9.0 Hz, H-7) and 6.83 (1H, d, *J* = 9.0 Hz, H-8), along with two pairs of methylene protons at *δ*_H_ 3.65 (1H, d, *J* = 11.4 Hz) and 2.72 (1H, d, *J* = 11.4 Hz) for H-5 and *δ*_H_ 2.50 (1H, dt, *J* = 14.4, 2.4 Hz) and 1.76 (1H, dt, *J* = 14.4, 2.4 Hz) for H-4. In addition, a dimethylamino group was observed at *δ*_H_ 2.63 (6H, s, 6-N(CH_3_)_2_), which was assigned to the D-ring dimethylamino substituent (C-6) [21,22]. These spectral features indicate that compound **2** retains the tetracycline scaffold comprising the B, C, and D rings. Consistent with the ^1^H NMR data, the ^13^C NMR and heteronuclear single quantum coherence (HSQC) spectra of compound **2** revealed carbon resonances attributable to the B, C, and D rings of minocycline at *δ*_C_ 200.1 (C-10), 173.8 (C-11), 160.7 (C-9), 144.9 (C-5a), 138.7 (C-6), 131.8 (C-7), 119.0 (C-9a), 117.2 (C-8), 102.0 (C-10a), 89.1 (C-11a), 67.8 (C-4a), 42.8 (6-N(CH_3_)_2_), 29.6 (C-4), and 29.4 (C-5) (Table 1). However, no ^1^H or ^13^C NMR signals corresponding to the A-ring functionalities of minocycline [21], including the A-ring dimethylamino group, amide, and olefinic moieties, were observed, indicating substantial structural modification or loss of the A ring (Figure 2A).

In addition, the ^1^H NMR spectrum displayed signals attributable to an oxygenated methine proton at *δ*_H_ 3.10 (1H, q, *J* = 1.2 Hz, H-3), a nitrogenated methine proton at *δ*_H_ 2.70 (1H, d, *J* = 1.2 Hz, H-2), and an additional methine proton at *δ*_H_ 3.71 (1H, dd, *J* = 2.4, 1.2 Hz, H-3a). Furthermore, the ^13^C NMR spectrum showed a characteristic signal corresponding to a ketone carbonyl carbon at *δ*_C_ 206.6 (C-1) and three methine carbons at *δ*_C_ 53.5 (C-3), 53.4 (C-3a), and 45.4 (C-2) (Table 1). The ^1^H–^1^H correlation spectra (COSY) of H-2/H-3/H-3a/H-4, along with the heteronuclear multiple bond correlations (HMBCs) from H-2 to C-1, C-3, and C-3a; from H-3 to C-1, C-2, C-3a, and C-11a; and from H-3a to C-1, C-2, C-3, C-4, and C-11a (Figure 2B), established the formation of a cyclopentanone-containing polycyclic scaffold, indicating that **2** is a cyclopenta[b]anthracene-type tetracycline derivative. The relative configuration of **2** was established based on the nuclear Overhauser effect spectroscopy (NOESY) correlations observed between H-2, H-3, and H-3a, along with small coupling constants (*J*_2,3,3a_ = 1.2 Hz), which confirmed a *cis*–*cis* arrangement. However, the absolute configuration of **2** could not be determined because of the lack of relevant reference circular dichroism (CD) or optical rotation data in the literature. Based on the above analyses, the planar structure of **2** was established as minocyclinosin A, a novel tetracycline analog featuring a cyclopenta[*β*]anthracene unit (Figure 2).

Radiation-based molecular transformations have been increasingly applied for the discovery of structurally diverse compounds; however, most previous studies have primarily focused on polyphenol-derived natural products [16,17,18,19]. In contrast, the present study demonstrates, for the first time that gamma irradiation can be effectively applied to a tetracycline-class antibiotic, leading to the generation of a structurally rearranged and previously unreported analog. Notably, gamma irradiation of minocycline (**1**) resulted in a dose-dependent molecular conversion, with 30 kGy irradiation exhibiting exceptionally high transformation efficiency, yielding minocyclinosin A (**2**) as the major product. This efficient one-step conversion highlights the potential utility of gamma irradiation as a practical and scalable approach for generating novel tetracycline derivatives. Given the widespread clinical use of tetracycline antibiotics and the structural diversity accessible through radiation-induced transformations, this strategy may provide a valuable platform for expanding the chemical space beyond conventional polyphenolic scaffolds and for discovering bioactive antibiotic-derived molecules with enhanced or differentiated biological properties.

### 2.2. Anti-Inflammatory Effects of Minocyclinosin A

**Figure 3:**
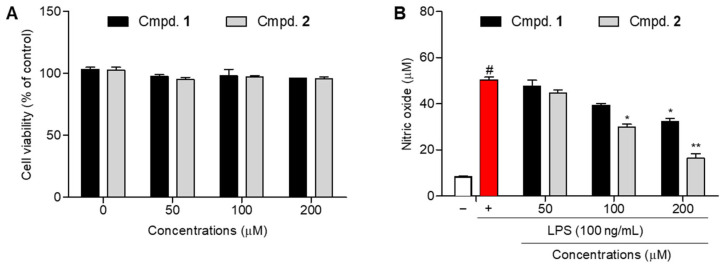
Effects of compounds **1** and **2** on anti-inflammatory activity in LPS-stimulated RAW 264.7 macrophages. (**A**) Cell viability determined by the MTT assay after treatment with compounds (50, 100, and 200 µM) for 24 h. (**B**) Cells were pretreated with compounds (50, 100, and 200 µM) for 1 h and then stimulated with LPS (100 ng/mL) for 24 h. Nitrite levels in the culture medium were measured using the Griess assay. Cells treated with neither compounds nor LPS were used as the control group. Data are expressed as mean ± SD (*n* = 3). ^#^ *p* < 0.05 represent significant differences compared to control group. * *p* < 0.05 and ** *p* < 0.01, represent significant differences comparted to only LPS-induced group. **1**: minocycline, **2**: minocyclinosin A.

NO, which is produced by inducible NO synthase in activated macrophages, is a critical mediator of inflammatory responses [23]. NO overproduction contributes to inflammatory tissue damage, and the suppression of NO generation in lipopolysaccharide (LPS)-stimulated macrophages is commonly used to assess anti-inflammatory activity [24]. Accordingly, the effects of the newly generated compounds on NO production were evaluated. Prior to evaluating their inhibitory effects on NO production, the cytotoxicity of the isolated compound (**2**) and parent compound (**1**) was assessed using an MTT assay. Cells were treated with compounds at concentrations of 50, 100, and 200 μM. Under all the tested conditions, both compounds exhibited cell viabilities exceeding 95%, indicating the absence of cytotoxic effects. Based on these results, subsequent NO inhibition experiments were performed within a non-cytotoxic concentration range (Figure 3A). In RAW 264.7 macrophages, stimulation with LPS markedly increased NO production. The accumulated NO level was 50.3 ± 2.1 μM, as determined by the Griess reagent assay [25], confirming successful induction of an inflammatory response. Treatment with minocycline (**1**) in LPS-stimulated RAW 264.7 macrophages resulted in NO production levels of 47.7 ± 4.3, 39.3 ± 1.3, and 32.2 ± 2.4 μM at concentrations of 50, 100, and 200 μM, respectively. Compared with the LPS-treated control, minocycline only exhibited a modest inhibitory effect, achieving an NO production inhibition of approximately 18 μM at the highest concentration tested, indicating relatively weak anti-inflammatory activity under these conditions. Conversely, minocyclinosin A markedly suppressed LPS-induced NO production in a concentration-dependent manner. NO levels were reduced to 44.7 ± 2.3, 29.9 ± 2.0, and 16.2 ± 3.5 μM at concentrations of 50, 100, and 200 μM, respectively. Notably, at 200 μM, minocyclinosin A demonstrated a more than twofold greater NO inhibitory efficacy compared with minocycline, highlighting a substantial enhancement of anti-inflammatory activity following gamma irradiation-induced structural transformation (Figure 3B).

Previous studies demonstrated that gamma irradiation can be applied to various natural products to enhance their biological properties via radiation-induced structural modification [16]. These irradiation processes induce diverse chemical reactions, including functionalization, structural rearrangement, and cyclization [17,26]. In this study, gamma irradiation of minocycline resulted in pronounced structural modification of the A ring, involving ring cleavage, degradation, and subsequent recyclization, ultimately forming a previously unreported tetracycline analog, minocyclinosin A (**2**). This radiation-induced molecular transformation was accompanied by a significant enhancement in the anti-inflammatory activity, as evidenced by the efficient suppression of LPS-induced NO production in RAW 264.7 macrophages. Notably, minocyclinosin A exhibited substantially stronger inhibitory activity against LPS-induced NO production than the parent compound, indicating a marked enhancement of anti-inflammatory potential following gamma irradiation-induced structural transformation. Collectively, these findings suggest that the radiation-induced modification of tetracycline antibiotics represents a promising strategy for expanding chemical diversity and generating bioactive derivatives with improved anti-inflammatory properties. This approach may provide valuable lead scaffolds for the development of therapeutic agents targeting inflammatory and immune-related diseases.

### 2.3. Antibacterial Effects of Minocyclinosin A

**Figure 4:**
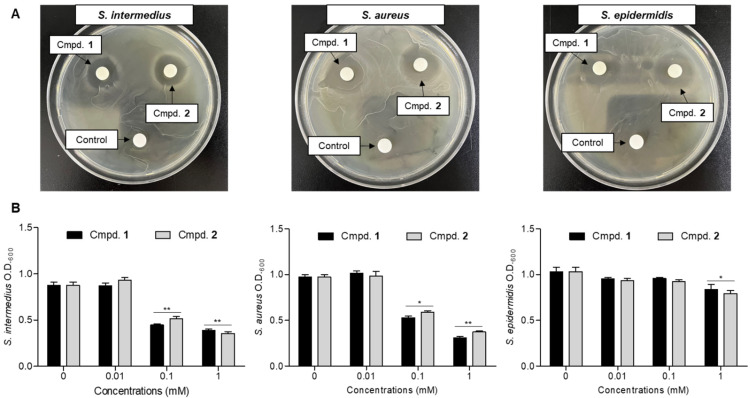
Antibacterial activity of compounds **1** and **2** against skin inflammation-associated *Staphylococcus* species. (**A**) Disk diffusion assay showing the inhibitory effects of compounds **1** and **2** (1 mM) against *Staphylococcus intermedius*, *S. aureus*, and *S. epidermidis*. Clear zones surrounding the disks indicate inhibition of bacterial growth. (**B**) Antibacterial rates of compounds **1** and **2** at concentrations of 0.01, 0.1, and 1 mM. The results are expressed as mean ± SD (*n* = 3). * *p* < 0.05 and ** *p* < 0.01, represent significant differences compared to control group. **1**: minocycline, **2**: minocyclinosin A.

*S. intermedius*, *S. aureus*, and *S. epidermidis* are Gram-positive bacteria closely associated with inflammatory skin conditions, ranging from pathogenic to opportunistic microorganisms [10,11,12]. These species are commonly used as models to evaluate antibacterial activity in skin-related inflammation studies. The antibacterial activities of minocycline (**1**) and the radiation-induced compound minocyclinosin A (**2**) were evaluated against *S. intermedius*, *S. aureus*, and *S. epidermidis*. Initial screening using a disk diffusion assay at a concentration of 1 mM showed that both compounds noticeably suppressed bacterial growth compared to the untreated control, as evidenced by the formation of clear inhibition zones (Figure 4A). Similar inhibitory patterns were observed across all three bacterial strains, indicating that the antibacterial activity of minocycline was retained after radiation-induced structural transformation. To further quantify antibacterial efficacy, antibacterial rates were measured at a concentration of 0.1 mM (Figure 4B). Under these conditions, both *S. intermedius* and *S. aureus* exhibited a nearly twofold reduction in bacterial growth relative to the untreated control. Notably, minocyclinosin A (**2**) displayed antibacterial activity comparable to that of the parent antibiotic minocycline (**1**), confirming that radiation-induced molecular modification did not adversely affect antibacterial potency.

The preservation of antibacterial activity observed for minocyclinosin A is particularly notable because of its enhanced anti-inflammatory effects in macrophages. While minocycline is primarily recognized for its antibiotic properties, radiation-induced structural transformation led to a newly generated derivative **2**, which exhibited markedly improved inhibition of inflammatory NO production (Figure 3) without compromising the antibacterial efficacy against skin inflammation-associated microorganisms. This dual profile is particularly relevant for skin-related inflammatory and allergic conditions, in which excessive immune activation and bacterial colonization often coexist. The comparable antibacterial activity of minocyclinosin A against *Staphylococcus* species and its enhanced anti-inflammatory potency suggest that the radiation-induced modification of tetracycline antibiotics may offer a promising strategy for developing multifunctional agents capable of simultaneously modulating inflammatory responses and controlling pathogenic or opportunistic skin bacteria.

In recent years, the clinical use of minocycline has declined owing to the availability of newer antibiotics with improved selectivity and reduced adverse effects. Nevertheless, minocycline remains a chemically distinctive tetracycline derivative with well-documented pleiotropic pharmacological properties, particularly its intrinsic anti-inflammatory and immunomodulatory activities. From a medicinal chemistry perspective, such structurally complex but underutilized antibiotics represent attractive substrates for molecular diversification. Accordingly, the present study was not intended to reposition minocycline as a frontline antimicrobial agent, but rather to evaluate the feasibility of γ-irradiation as a transformation platform for upgrading clinically declining drugs. The successful conversion of minocycline into minocyclinosin A, which retained antibacterial activity while exhibiting markedly enhanced anti-inflammatory efficacy, demonstrates that radiation-induced modification can selectively improve functional profiles without abolishing the original pharmacological scaffold. This finding supports the concept that radiation chemistry may provide a practical route for revitalizing legacy pharmaceuticals and generating multifunctional derivatives with therapeutic potential beyond their original indications.

### 2.4. Comparative HPLC Analysis of Minocyclinosin A

**Figure 5:**
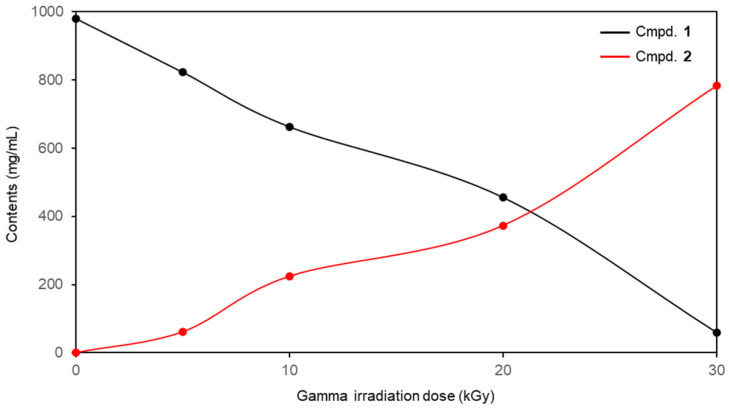
Contents (mg/g) of individual components in the γ-irradiated minocycline solutions at different irradiation doses. **1**: minocycline, **2**: minocyclinosin A.

Quantitative evaluation of the radiation-induced compounds was performed using minocycline samples exposed to gamma irradiation at doses of 5, 10, 20, and 30 kGy using an external standard-based HPLC method. The quantitative results are summarized in Figure 1 and Figure 5. Calibration curves were established using five independently prepared concentration levels (*n* = 5), all of which demonstrated excellent linearity with correlation coefficients exceeding 0.999. The retention times of minocyclinosin A (**2**) and minocycline (**1**) were observed at *t*_R_ 12.3 and 5.4 min, respectively. Quantitative HPLC analysis revealed that the content of minocyclinosin A (**2**) in the irradiated reaction mixtures increased in a dose-dependent manner, with concentrations of 61.1 ± 0.1, 224.5 ± 0.9, 373.9 ± 1.1, and 783.0 ± 1.0 mg/g obtained at irradiation doses of 5, 10, 20, and 30 kGy, respectively (Figure 5). Conversely, the residual amount of minocycline (**1**) was progressively reduced as the irradiation dose increased, supporting the efficient conversion of the parent compound into the newly generated product. Collectively, these results demonstrate that gamma irradiation effectively induces the molecular transformation of minocycline, with irradiation at 30 kGy providing the highest accumulation of minocyclinosin A (**2**) under the experimental conditions employed.

Minocyclinosin A exhibited markedly enhanced anti-inflammatory activity compared with minocycline, while maintaining comparable antibacterial activity against skin inflammation-associated bacteria. This balance between improved anti-inflammatory efficacy and preserved antibacterial function is particularly noteworthy, as it suggests that radiation-induced structural modifications can selectively enhance biological properties without compromising the original antibiotic activity. Notably, gamma irradiation at 30 kGy afforded a high conversion yield of approximately 78.3%, indicating an efficient and practical transformation process. Given the high conversion efficiency and favorable biological profile of minocyclinosin A, this radiation-based approach offers a promising strategy for expanding the applicability of tetracycline-derived compounds to diverse therapeutic and dermatological contexts.

## 3. Materials and Methods

### 3.1. Chemicals and Instruments

Minocycline, acetonitrile, methanol, formic acid (HPLC grade), CD_3_OD, LPS, and the Griess reagent were obtained from Sigma-Aldrich (St. Louis, MO, USA). All the other chemicals were of analytical grade. 1D- and 2D-NMR spectra were recorded on a Bruker Avance NEO-600 spectrometer (Bruker, Karlsruhe, Germany) at 600 and 150 MHz, respectively, with chemical shifts (*δ*, ppm) referenced to CD_3_OD (*δ*_H_ 3.31, *δ*_C_ 49.0). ESI mass spectra were acquired using a Vanquish UPLC system (Thermo Fisher Scientific, Waltham, MA, USA). UV spectra and optical rotations were measured using T-60 (PG Instruments, Leicestershire, UK) and P-2000 spectrometers (JASCO, Tokyo, Japan), respectively. Column chromatography was performed on Toyopearl HW-40 (coarse grade; Tosoh Co., Tokyo, Japan) and YMC-gel ODS AQ 120-50S (50 μm; YMC Co., Kyoto, Japan). Absorbance was measured using a microplate reader (Infinite F200, Tecan Austria GmBH, Grodig, Austria).

### 3.2. Gamma Irradiation Procedure

Gamma irradiation was carried out at 24 °C using a cobalt-60 irradiator (point source AELC, IR-79, MDS Nordion International Co., Ltd., Ottawa, ON, Canada) at the Advanced Radiation Technology Institute, Korea Atomic Energy Research Institute (Jeongeup, Republic of Korea). The source strength was approximately 320 kCi at a dose rate of 10 kGy/h [17]. Pure minocycline (500 mg) was dissolved in methanol (500 mL) in a sealed glass bottle and irradiated at 5, 10, 20, and 30 kGy. After irradiation, each reaction mixture was immediately concentrated under reduced pressure to remove the solvent and dried prior to further analysis.

### 3.3. Determination of the Newly Generated Products

The gamma-irradiated samples were analyzed by HPLC to monitor radiation-induced molecular transformations. HPLC analysis was performed using an Agilent 1200 HPLC system equipped with a photodiode array (PDA) detector (Agilent Technologies, Palo Alto, CA, USA) and a YMC-Pack ODS A-302 column (4.6 mm i.d. × 150 mm, YMC Co., Kyoto, Japan). The mobile phase consisted of a linear gradient of 1% formic acid in water and acetonitrile, increasing to acetonitrile over 30 min, at a flow rate of 1.0 mL/min. UV detection was carried out at 280 nm, and the column temperature was maintained at 40 °C.

HPLC results revealed the appearance of a newly generated peak at a retention time of 12.3 min in the irradiated samples, while the peak corresponding to the parent minocycline gradually decreased with increasing irradiation dose. The parent compound was no longer detectable in the sample irradiated at 30 kGy, and the newly generated peak was observed as the predominant component (Figure 1). Based on these results, the sample irradiated at 30 kGy was selected for isolation and purification.

### 3.4. Isolation and Structural Elucidation of the Generated Products

The gamma-irradiated reaction mixture (0.5 g) was initially subjected to flash column chromatography using a Toyopearl HW-40 column (2.5 cm i.d. × 40 cm). Elution was performed using a stepwise gradient of H_2_O/MeOH (100:0 to 20:80, *v*/*v*), followed by the addition of 70% aqueous acetone to obtain six subfractions (IMC01–IMC06). Among these fractions, subfraction IMC03 (77.5 mg), which contained the newly generated product, was further purified using reversed-phase column chromatography on a YMC GEL ODS AQ 120-50S column (1.5 cm i.d. × 40 cm). Elution with 70% methanol in water yielded **2** as a pure compound (52.9 mg) at a retention time of 12.3 min (Figure S1).

Minocyclinosin A (**2**): Yellow amorphous powder, [α]_D_ −269.9 (*c* 0.1, MeOH); UV _λmax_ MeOH nm (log ε): 206 (1.17), 265 (1.54) nm; ^1^H and ^13^C NMR, see Table 1; ESIMS *m*/*z* 413 [M + Na]^+^, HRESIMS *m*/*z* 413.1336 [M + Na]^+^ (calculated for C_19_H_22_O_7_N_2_Na, 413.1319) (Figures S2–S8).

### 3.5. Cell Culture

The RAW 264.7 mouse monocyte macrophage cell line (KCLB No. 40071) was obtained from the Korean Cell Line Bank (Seoul, Republic of Korea). Cells were cultured under at 37 °C in a humidified 5% CO_2_ atmosphere in Dulbecco’s Modified Eagle’s Medium (DMEM) supplemented with 10% fetal bovine serum and 1% penicillin/streptomycin.

### 3.6. Cell Viability Assay

Cell viability of RAW 264.7 cells was assessed using the MTT assay [27]. Cells were seeded at 5 × 10^4^ cells/well in 96-well plates and incubated for 24 h at 37 °C. They were then treated with compounds **1** and **2** (50, 100, and 200 μM) for 24 h in serum-free medium. Subsequently, 0.5 mg/mL MTT solution in DMEM was added to each well, and the plates were incubated for 3 h at 37 °C. Formazan crystals were dissolved in DMSO, and the absorbance was measured at 570 nm. The cell viability was determined relative to the control group (100%).

### 3.7. Nitric Oxide (NO) Assay

RAW 264.7 cells (5 × 10^4^/well) were plated in a 96-well plate and incubated for 24 h at 37 °C. They were then pretreated with various concentrations of compounds **1** and **2** (50, 100, and 200 μM) for 2 h before LPS (100 ng/mL) stimulation for 24 h at 37 °C. NO production was measured using the Griess reaction (Griess reagent). The culture supernatant (100 μL) was mixed with the Griess reagent (100 μL) at room temperature for 20 min. The absorbance of the reaction mixture was measured at 548 nm to determine the NO levels [25].

### 3.8. Antibacterial Effects

The antibacterial activities of minocycline (**1**) and minocyclinosin A (**2**) were evaluated using a broth microdilution susceptibility assay. *S. intermedius* (KCTC 3344), *S. aureus* (KCTC 1621), and *S. epidermidis* (KCTC 3958) were obtained from the Korean Collection for Type Cultures (KCTC), Korea Research Institute of Bioscience and Biotechnology. Each bacterial strain was cultured overnight in Luria–Bertani (LB) broth at 37 °C and adjusted to the appropriate cell density prior to use. In a 96-well microplate, 100 µL of LB broth was added to each well, followed by the addition of test compounds prepared at the desired concentrations. Serial dilutions were performed to obtain final test concentrations (0.01, 0.1, and 1mM). Subsequently, 100 µL of the bacterial suspension was added to each well. The plates were incubated at 37 °C for 24 h, and all experiments were conducted in triplicate. After incubation, the bacterial growth was assessed by measuring the optical density at 600 nm using a microplate reader. Antibacterial activity was determined by comparing bacterial growth in the treated wells with that of the untreated control [28].

Sterile paper disks (6 mm in diameter) were impregnated with the test compounds at a concentration of 1 mM and placed on the inoculated agar surfaces. Disks containing the solvents were used as negative controls. The plates were incubated at 37 °C for 24 h, after which antibacterial activity was assessed by measuring the diameter of the inhibition zones surrounding each disk [29]. All experiments were performed in triplicate, and the results were expressed as the mean inhibition zone diameter.

### 3.9. Quantitation of Minocyclinosin A

Quantitative analysis of minocycline (**1**) and the radiation-induced product minocyclinosin A (**2**) was performed using an Agilent 1200 HPLC system equipped with a photodiode array (PDA) detector (Agilent Technologies, Palo Alto, CA, USA). Chromatographic separation was achieved on a reversed-phase YMC-Pack ODS-A column (4.6 mm i.d. × 150 mm, 5 μm; YMC Co., Kyoto, Japan). The mobile phase consisted of solvent A (0.1% formic acid in water) and solvent B (acetonitrile) delivered using a linear gradient from 10% to 100% solvent B over 30 min. The flow rate was set at 1.0 mL/min, and detection was performed at a wavelength of 280 nm using the PDA detector. The identification of minocycline and minocycline A in the irradiated samples was based on a comparison of their retention times with those of authentic standards. Stock solutions of minocycline and minocyclinosin A were prepared in methanol at a concentration of 5000 mg/L. Working standard solutions were prepared by serial dilution with methanol to obtain five concentration levels ranging from 31.25 to 500 mg/mL. Prior to injection, all standard and sample solutions were filtered through syringe filters (Fisher Scientific, Fair Lawn, MJ, USA). Calibration curves were constructed by plotting integrated peak areas against the corresponding concentrations of each standard. Linearity was evaluated by linear regression analysis, which demonstrated excellent correlation over the tested concentration range (Figure S9) [17].

### 3.10. Statistical Analysis

All data were evaluated by one-way analysis of variance (ANOVA), followed by Duncan’s multiple range test, and the results were considered statistically significant when the *p*-value was <0.05. Experiments were independently performed at least three times.

## 4. Conclusions

In this study, gamma irradiation was applied to minocycline (**1**) to induce a molecular transformation, resulting in the isolation and structural elucidation of minocyclinosin A (**2**), a previously unreported tetracycline analog. Spectroscopic analyses revealed extensive A-ring cleavage and recyclization, leading to the formation of a unique cyclopenta[b]anthracene-containing polycyclic scaffold. The transformation proceeded in a dose-dependent manner under 30 kGy irradiation, affording a high conversion efficiency of approximately 78.3% and demonstrating the practicality of this one-step radiation-based approach. Biological evaluation showed that minocyclinosin A exhibited significantly enhanced anti-inflammatory activity, as evidenced by the effective inhibition of LPS-induced NO production in RAW 264.7 macrophages, while retaining antibacterial activity comparable to that of minocycline (**1**) against skin inflammation-associated *Staphylococcus* species. These findings indicate that gamma irradiation enables the selective enhancement of anti-inflammatory properties without compromising the intrinsic antibiotic function of the tetracycline scaffold. This study highlights the potential of radiation-induced molecular transformation as a viable strategy for generating multifunctional antibiotic-derived compounds applicable to inflammatory and immune-related disorders, particularly in dermatological settings.

## Figures and Tables

**Table 1:** ^1^H and ^13^C NMR data of compound **2** ^1^.

Positions	*δ*_H_ (*J* in Hz) ^2^	*δ*_C_, Type ^3^
1	-	206.6, C
2	2.70 (d, 1.2)	45.4, CH
3	3.10 (q, 1.2)	53.5, CH
3a	3.71 (dd, 2.4,1.2)	53.4, CH
4	2.50 (dt, 14.4, 2.4), 1.76 (dt, 14.4, 2.4)	29.6, CH_2_
4a	-	67.8, C
5	3.65 (d, 11.4), 2.72 (d, 11.4)	29.4, CH_2_
5a	-	144.9, C
6	-	138.7, C
7	7.50 (d, 9.0)	131.8, CH
8	6.83 (d, 9.0)	117.2, CH
9	-	160.7, C
9a	-	119.0, C
10	-	200.1, C
10a	-	102.0, C
11	-	173.8, C
11a	-	89.1, C
6-N(CH_3_)_2_	2.63 (s)	42.8, CH_3_

^1^ Measured in CD_3_OD, and assignments of chemical shifts were based on the analysis of 1D and 2D NMR spectra. Overlapping signals were assigned from the HSQC, HMBC, and ^1^H-^1^H COSY spectra without designating the multiplicity. ^2^ Data (*δ*) measured at 600 MHz. ^3^ Data (*δ*) measured at 150 MHz.

## Data Availability

The data presented in this study are available on request from the corresponding authors.

## Supplementary Materials

The following are available online at https://www.mdpi.com/article/10.3390/molecules31030435/s1, Figure S1: HPLC chromatograms of isolated compound **2**. Figure S2: ^1^H NMR spectrum of **2** in CD_3_OD. Figure S3: ^13^C NMR spectrum of **2** in CD_3_OD. Figure S4: HSQC spectrum of **2** in CD_3_OD. Figure S5: HMBC spectrum of **2** in CD_3_OD. Figure S6: ^1^H-^1^H COSY spectrum of **2** in CD_3_OD. Figure S7: NOESY spectrum of **2** in CD_3_OD. Figure S8: HRESIMS spectrum of **2**. Figure S9: Calibration curves of minocycline (**1**) and minocyclinosin A (**2**).
