# Supplementary material for: Radiation-Induced Synthesis of a Minocycline-Derived Polycyclic Scaffold with Anti-Inflammatory and Antibacterial Effects
Source: Molecules. 2026 Jan 27;31(3):435. doi: 10.3390/molecules31030435 (PMC12899902; doi:10.3390/molecules31030435)
Supplement: Supplementary file 1 [file molecules-31-00435-s001.zip › molecules-4108434-supplementary.pdf]

Supporting Information

Radiation-Induced Synthesis of a Minocycline-Derived Polycyclic Scaffold with Anti-Inflammatory and Antibacterial Effects

Gyeong Han Jeong [1,†], Hanui Lee [1,†], Tae Hoon Kim [2], Byung Yeoup Chung [1], Seung Sik Lee [1,3,*] and Hyoung-Woo Bai [1,3,*]

[1] Advanced Radiation Technology Institute (ARTI), Korea Atomic Energy Research Institute (KAERI), Jeongeup 56212, Republic of Korea; jkh4598@kaeri.re.kr (G.H.J.); hnlee11@kaeri.re.kr (H.L.); bychung@kaeri.re.kr (B.Y.C.)

[2] Department of Food Science and Biotechnology, Daegu University, Gyeongsan 38453, Republic of Korea; skyey7@daegu.ac.kr

[3] Department of Radiation Science, University of Science and Technology (UST), Daejeon 34113, Republic of Korea

* Correspondence: sslee@kaeri.re.kr (S.S.L.); hbai@kaeri.re.kr (H.-W.B.)

† These authors contributed equally to this work.

Contents

Figure S1. HPLC chromatograms of isolated compound **2**.

Figure S2. ^{1}H NMR spectrum of **2** in CD_3OD.

Figure S3. ^{13}C NMR spectrum of **2** in CD_3OD.

Figure S4. HSQC spectrum of **2** in CD_3OD.

Figure S5. HMBC spectrum of **2** in CD_3OD.

Figure S6. ^{1}H-^{1}H COSY spectrum of **2** in CD_3OD.

Figure S7. NOESY spectrum of **2** in CD_3OD.

Figure S8. HRESIMS spectrum of **2**.

Figure S9. Calibration curves of minocycline (**1**) and minocyclinosin A (**2**).

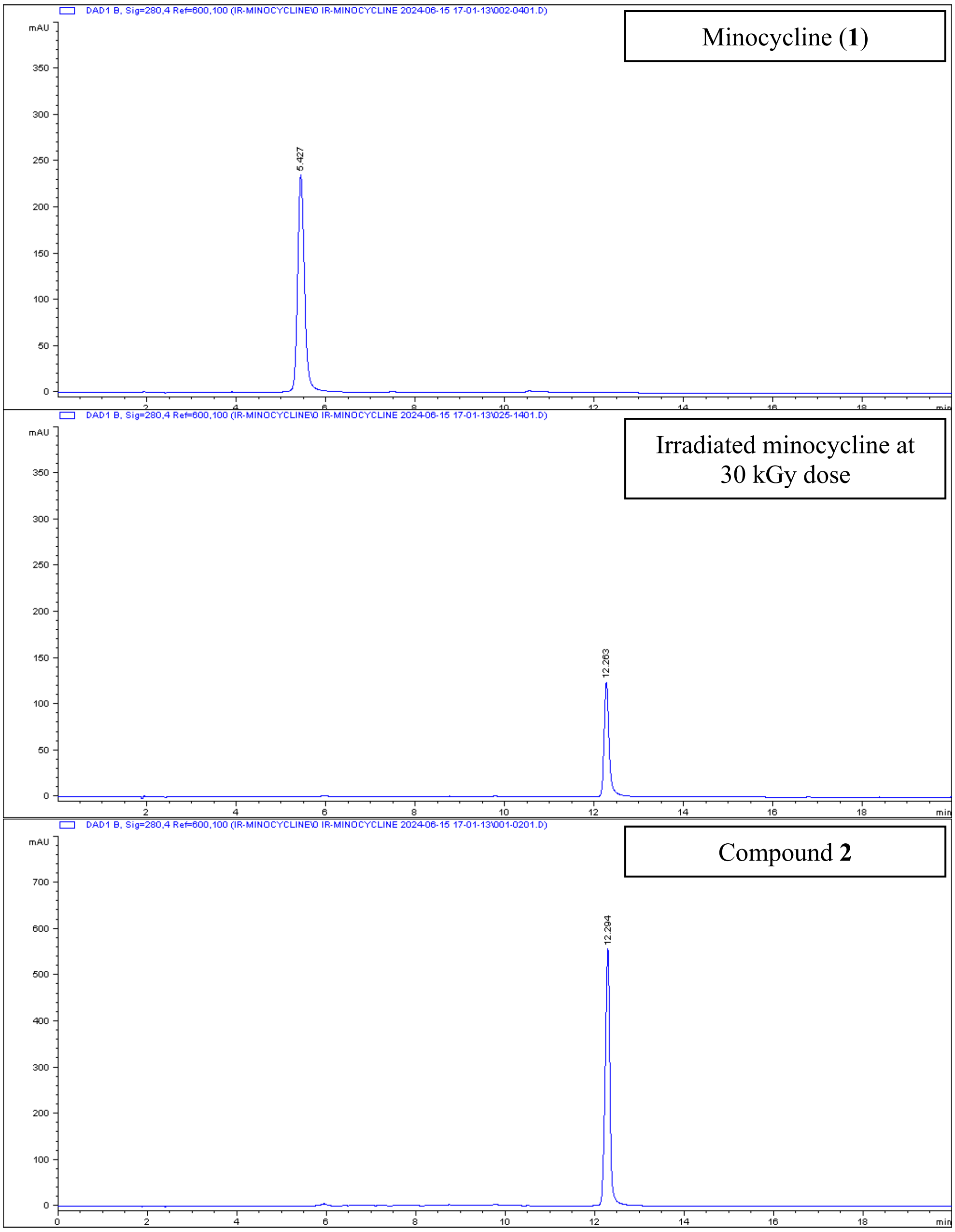

Figure S1. HPLC chromatograms of isolated compound.

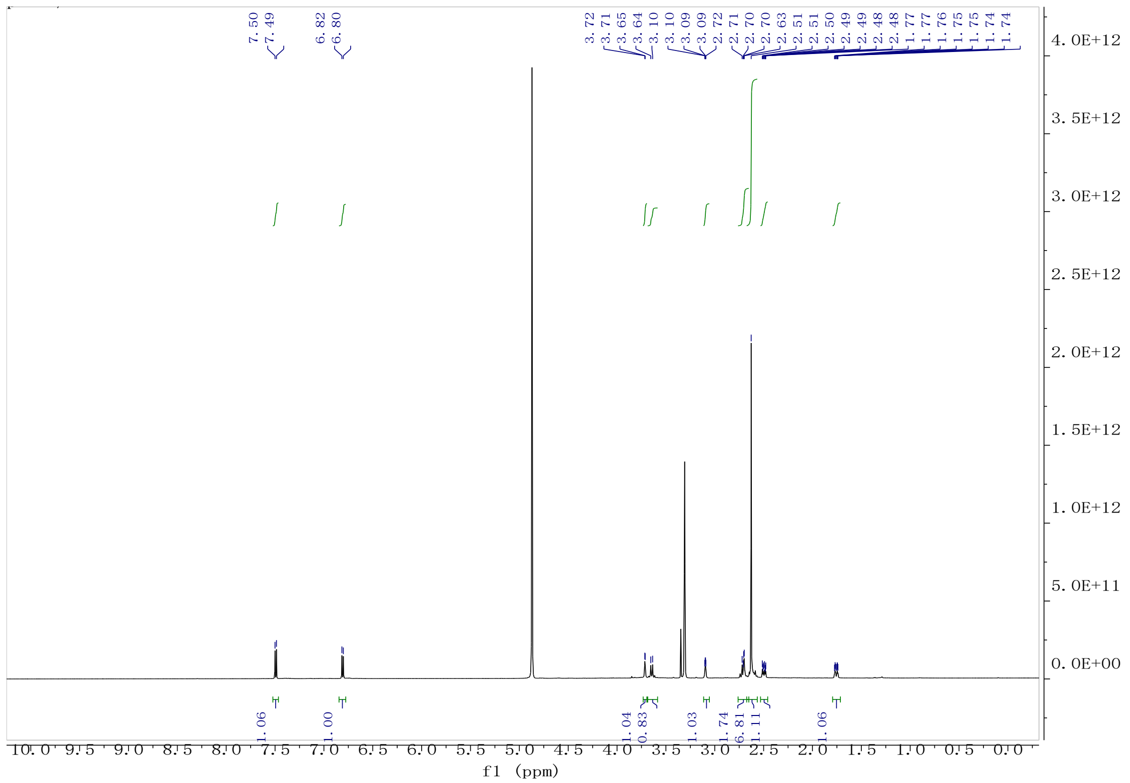

Figure S2. ^{1}H NMR spectrum of **2** in CD_3OD.

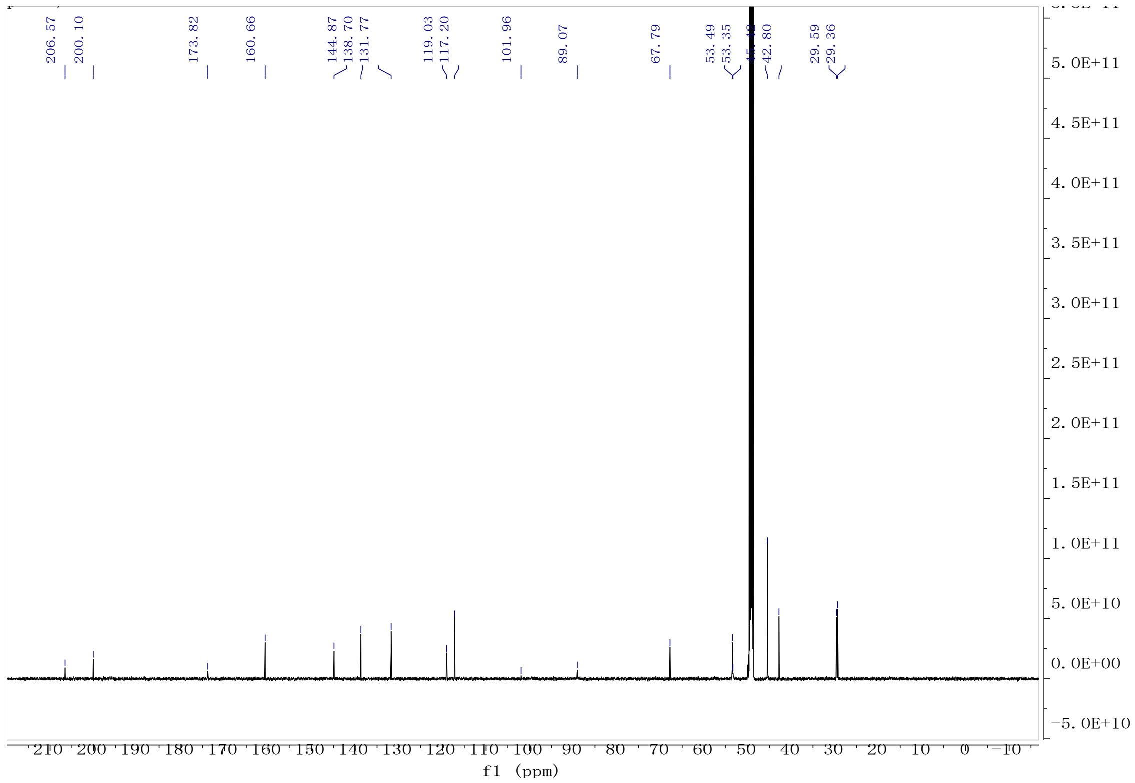

Figure S3. ^{13}C NMR spectrum of **2** in CD_3OD.

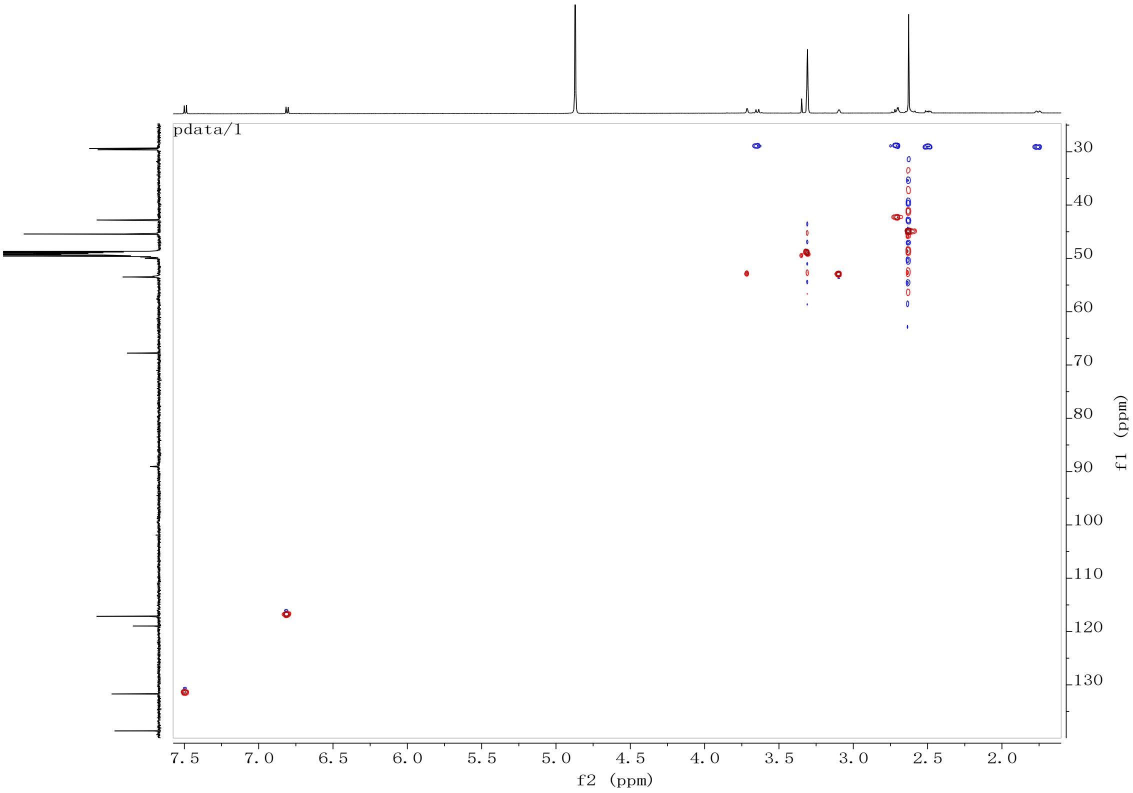

Figure S4. HSQC spectrum of **2** in CD_3OD.

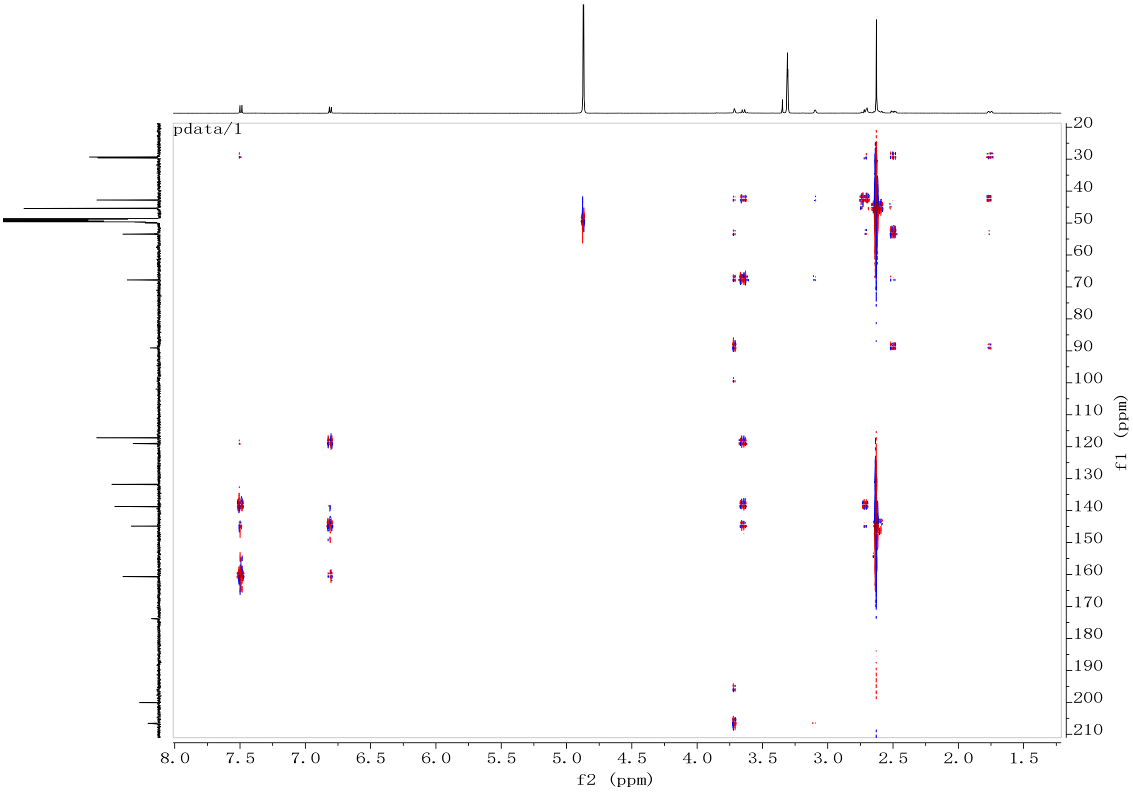

Figure S5. HMBC spectrum of **2** in CD_3OD.

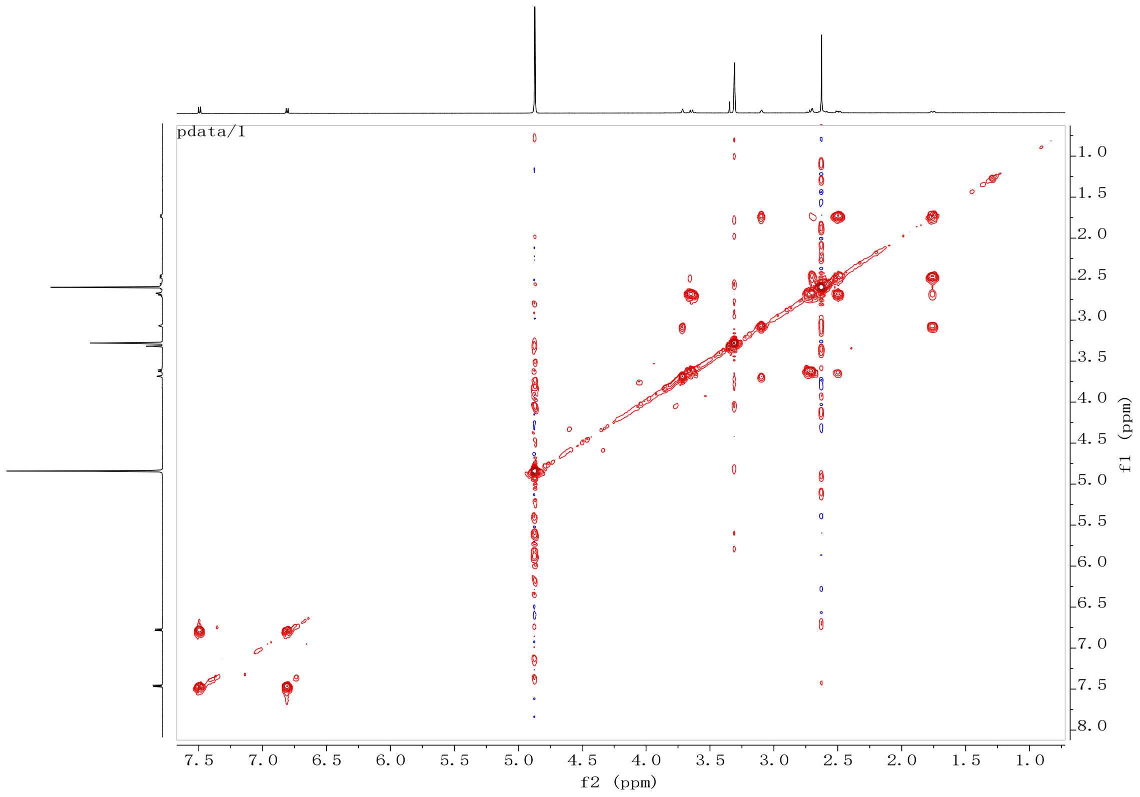

Figure S6. ^{1}H-^{1}H COSY spectrum of **2** in CD_3OD.

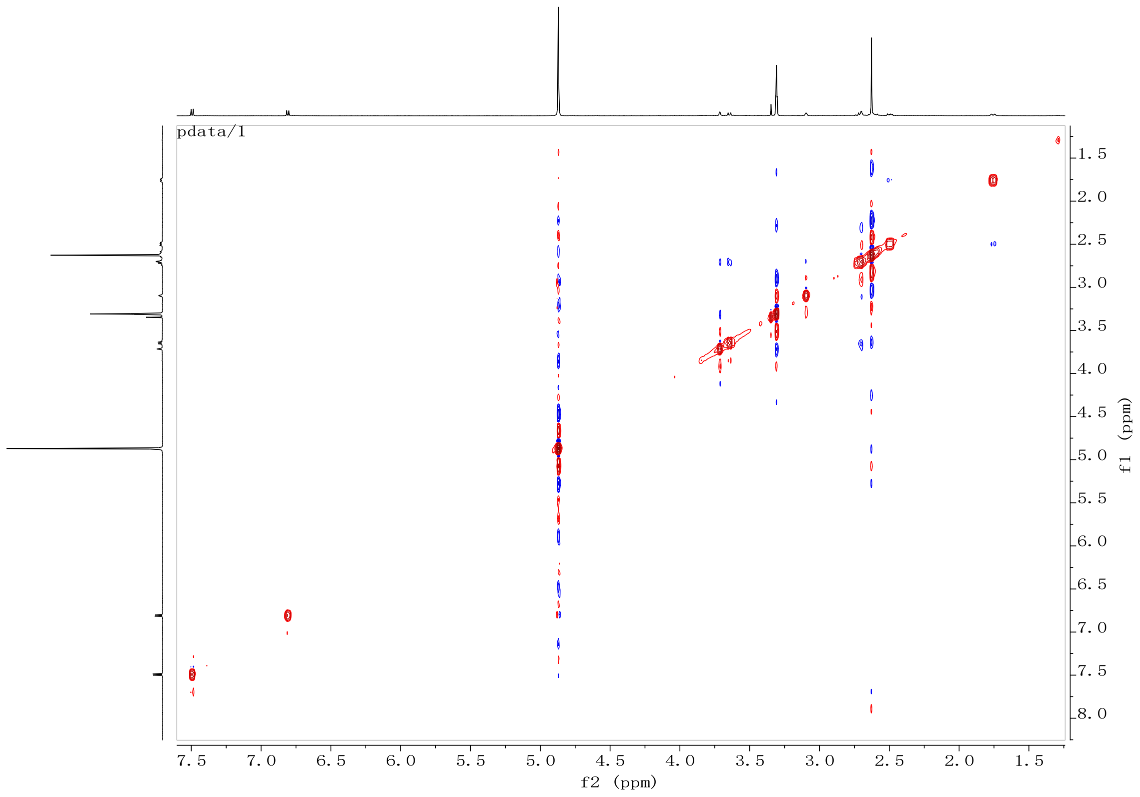

Figure S7. NOESY spectrum of **2** in CD_3OD.

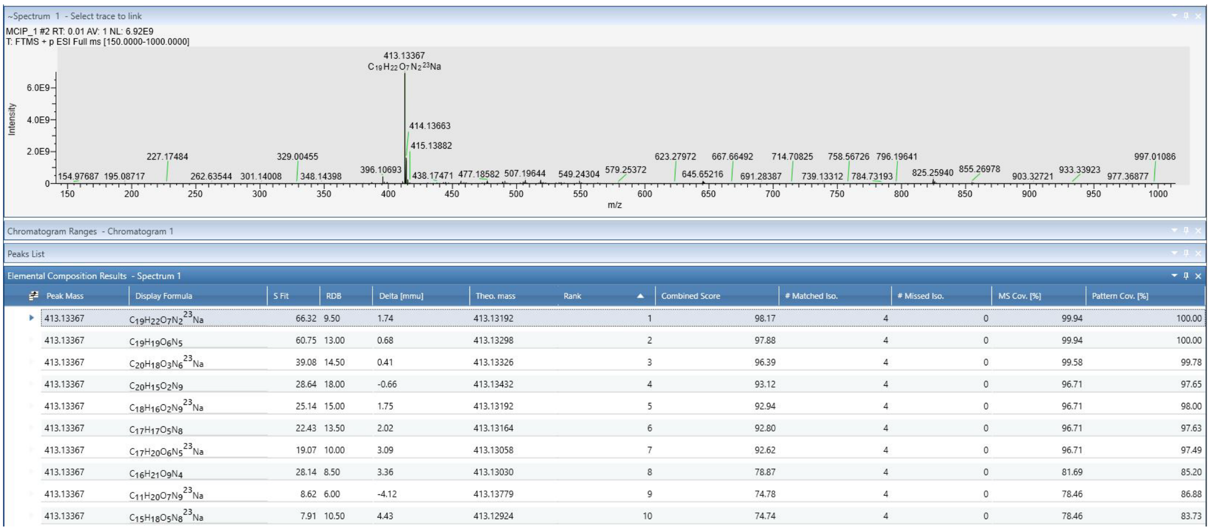

Peak Mass	Display Formula	S Fit	RDB	Delta [mmu]	Theo. mass	Rank	Combined Score	# Matched Iso.	# Missed Iso.	MS Cov. [%]	Pattern Cov. [%]
413.13367	$C_{19}H_{22}O_7N_2{}^{23}Na$	66.32	9.50	1.74	413.13192	1	98.17	4	0	99.94	100.00
413.13367	$C_{19}H_{19}O_6N_5$	60.75	13.00	0.68	413.13298	2	97.88	4	0	99.94	100.00
413.13367	$C_{20}H_{18}O_3N_6{}^{23}Na$	39.08	14.50	0.41	413.13326	3	96.39	4	0	99.58	99.78
413.13367	$C_{20}H_{15}O_2N_9$	28.64	18.00	-0.66	413.13432	4	93.12	4	0	96.71	97.65
413.13367	$C_{18}H_{16}O_2N_9{}^{23}Na$	25.14	15.00	1.75	413.13192	5	92.94	4	0	96.71	98.00
413.13367	$C_{17}H_{17}O_5N_8$	22.43	13.50	2.02	413.13164	6	92.80	4	0	96.71	97.63
413.13367	$C_{17}H_{20}O_6N_5{}^{23}Na$	19.07	10.00	3.09	413.13058	7	92.62	4	0	96.71	97.49
413.13367	$C_{16}H_{21}O_9N_4$	28.14	8.50	3.36	413.13030	8	78.87	4	0	81.69	85.20
413.13367	$C_{11}H_{20}O_7N_9{}^{23}Na$	8.62	6.00	-4.12	413.13779	9	74.78	4	0	78.46	86.88
413.13367	$C_{15}H_{18}O_5N_8{}^{23}Na$	7.91	10.50	4.43	413.12924	10	74.74	4	0	78.46	83.73

Figure S8. HRESIMS spectrum of **2**.

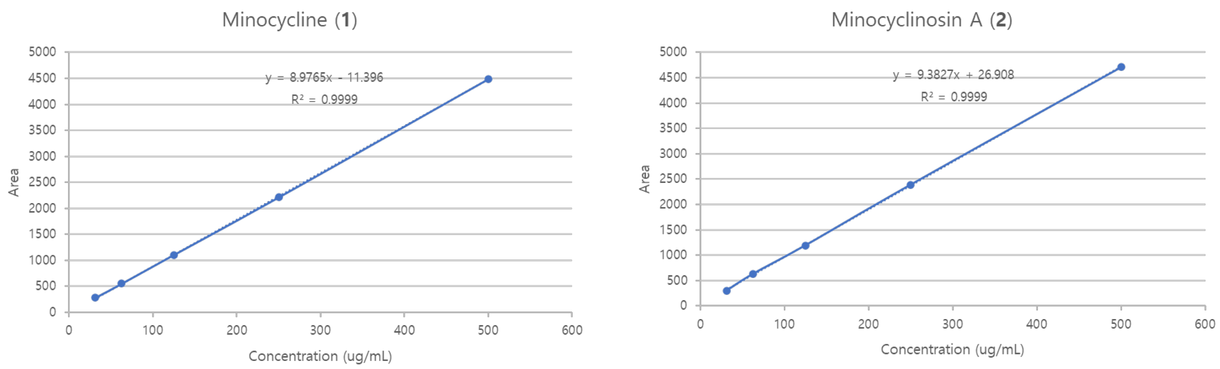

Figure S9. Calibration curves of minocycline (**1**) and minocyclinosin A (**2**).
